# Regional differences in recombination hotspots between two chicken populations

**DOI:** 10.1186/1471-2156-11-11

**Published:** 2010-02-08

**Authors:** Martin G Elferink, Pieter van As, Tineke Veenendaal, Richard PMA Crooijmans, Martien AM Groenen

**Affiliations:** 1 Animal Breeding and Genomics Centre, Wageningen University and Research Centre, PO Box 338, 6700 AH Wageningen, the Netherlands

## Abstract

**Background:**

Although several genetic linkage maps of the chicken genome have been published, the resolution of these maps is limited and does not allow the precise identification of recombination hotspots. The availability of more than 3.2 million SNPs in the chicken genome and the recent advances in high throughput genotyping techniques enabled us to increase marker density for the construction of a high-resolution linkage map of the chicken genome. This high-resolution linkage map allowed us to study recombination hotspots across the genome between two chicken populations: a purebred broiler line and a broiler × broiler cross. In total, 1,619 animals from the two different broiler populations were genotyped with 17,790 SNPs.

**Results:**

The resulting linkage map comprises 13,340 SNPs. Although 360 polymorphic SNPs that had not been assigned to a known chromosome on chicken genome build WASHUC2 were included in this study, no new linkage groups were found. The resulting linkage map is composed of 31 linkage groups, with a total length of 3,054 cM for the sex-average map of the combined population. The sex-average linkage map of the purebred broiler line is 686 cM smaller than the linkage map of the broiler × broiler cross.

**Conclusions:**

In this study, we present a linkage map of the chicken genome at a substantially higher resolution than previously published linkage maps. Regional differences in recombination hotspots between the two mapping populations were observed in several chromosomes near the telomere of the p arm; the sex-specific analysis revealed that these regional differences were mainly caused by female-specific recombination hotspots in the broiler × broiler cross.

## Background

Genetic linkage maps are essential to identify genomic regions that influence complex phenotypes (quantitative trait loci), to assist in the sequence assembly of genomes, and to study recombination across the genome. Linkage analysis and genome-wide association studies not only require high marker densities, but also accurate linkage maps in order to detect quantitative trait loci [1].

High-density linkage maps have been described for humans [2-4], mice [5], rats [6], and chickens [7]. Chicken linkage maps have been published ranging from 100 RFLP markers [8] to a high-density map comprising thousands of markers, most of which are single nucleotide polymorphisms (SNP) [7].

In combination with a physical BAC contig map [9], linkage maps of the chicken [10-12] were used to construct the draft genome sequence of the chicken. The draft sequence of the chicken genome, published in 2004, comprises 1.05 Gb [13]. In chicken genome build WASHUC2 (May 2006) there were a total of 997 Mb of assigned sequences, which covered the two sex chromosomes (Z and W) and 29 of the 38 autosomes. The unassembled sequences that remained were combined in chromosome unassigned. The most recent linkage map, published in 2009 by Groenen *et al*., consists of 34 different linkage groups (including GGZ); thus, at least five autosomal chromosomes are still entirely unrepresented [7].

Differences in the sizes of the linkage map were found among several chicken populations [7,10,11,14,15]. In these studies, domesticated populations tended to have increased recombination compared to nondomesticated populations. This finding was in agreement with the hypothesis that selection leads to higher rates of recombination [16]. Due to the limited resolution of the published chicken linkage maps the specific underlying regions where recombination differs among the chicken populations could not be identified. Moreover, these studies mainly focused on sex-average recombination, and did not take into account the influence of sex on recombination in chickens.

The availability of more than 3.2 million SNPs in the chicken genome (dbSNP build 128 and [17]) and the recent advances in high-throughput genotyping techniques makes it feasible to increase marker density for linkage analysis and genome-wide association studies and to study recombination rates across the genome in the chicken.

In this study, we present a high-resolution linkage map of the chicken genome based on data from a cross between two different broiler lines (*n *= 306) and on data from a different single purebred broiler line (*n *= 1313). Both populations were genotyped with an 18K SNP Illumina Infinium iSelect Beadchip. The high-resolution linkage maps generated in this study allowed us to study regions of recombination hotspots between the two mapping populations and between the sexes.

## Methods

### Marker selection

In total, 17,790 markers were included on the Illumina Infinium iSelect Beadchip (Additional File 1). Markers were selected from dbSNP build 122. The Beadchip consisted of 17,177 markers that had been mapped and 613 markers that had not been mapped to a chromosome or linkage group. Markers were distributed evenly across each chromosome, with marker densities based on the size of the chromosome. For GGA1-GGA5 and GGZ, markers were selected every 50 kb; for GGA6-GGA10 every 36 kb; for GGA11-GGA20 every 25 kb; and for GGA21-GGA28 every 15.5 kb. Two additional linkage groups, which were not assigned to a chromosome, were also included on the beadchip: LGE22C19W28_E50C23 (from here on called LGE22) and LGE64. The unmapped markers were located on contigs larger than 100,000 bp, which were found in the unassigned sequences of the draft sequence (chromosome unassigned). The 613 markers were selected randomly, except for the size of the contig in which they were located.

Genotyping was performed using the standard protocol for Infinium iSelect Beadchips. Data were analyzed with Beadstudio Genotyping v3.0.19.0, and quality control was performed according to the guidelines from the Infinium genotyping data analysis protocol [18].

### Populations

In total, 1,619 animals from two populations were genotyped with the 18K SNP beadchip. Blood and DNA sample collection was carried out by licensed and authorized personnel under approval of Hendrix Genetics. Population 1 was an advanced intercross line derived from a cross between two broiler dam lines [19,20]. The maternal line was selected for reproduction (egg numbers as the most important trait, as well as hatching of fertile eggs) and, to a lesser extend, body weight. The maternal line was not selected for feed conversion rate and breast meat percentage. The paternal line was selected for growth and feed conversion rate (almost equally important), and selection with regard to reproduction was performed to keep performance constant (it also compensated the negative effects of selection for growth). The paternal line, moreover, was also subject to some selection for conformation. There was no selection for breast meat percentage for this line. The maternal and paternal lines both originated from the White Plymouth Rock breed. Population 1 was used previously for quantitative trait loci mapping of pulmonary hypertension syndrome [19,21]; fatness traits in broilers [22]; and bodyweight, growth rate and feed efficiency [23,24]. Combined with other populations, a subset of population 1 has previously been used to construct the consensus linkage map of the chicken genome [7,12]. In total, 306 animals were genotyped from population 1: 10 full-sib families of generation 1; 20 parents (10 males and 10 females) and 50 offspring (11 males and 39 females); and 37 full- and half-sib families of generation 6 or 7; 66 parents (32 males and 34 females) and 170 offspring (61 males, 67 females, and 42 of unknown sex). Population 2 consisted of a third purebred commercial broiler dam line that was selected for breast meat percentage. This population also originated from the White Plymouth Rock breed. In total, 1,313 animals were genotyped from population 2: 266 parents (68 males and 198 females) and 1,047 offspring (107 males and 940 females).

### Linkage analysis

The linkage map was constructed with a modified version of CRI-MAP [25]. This modified version can handle large datasets and was provided by Drs. Liu and Grosz of Monsanto Company (St. Louis, MO, USA). During construction of the linkage map, a marker was considered to be informative if it had at least 20 informative meioses. The linkage map was constructed with the use of five options: AUTOGROUP, BUILD, CHROMPIC, FLIPSN, and FIXED. AUTOGROUP was used to check each chromosome unassigned marker for linkage to a known chromosomes or linkage groups (thresholds used: LOD = 4, informative meioses = 0, different chromosomes = 5, and linkage ratio = 0.5). Markers were assigned to a specific chromosome if linkage was found, or remained in chromosome unassigned if no linkage was found. The initial marker order was similar to the order in which the markers were located on the physical map (WASHUC2 build, May 2006). The BUILD option was used to determine the most likely position of the newly assigned markers in the marker order. Markers were mapped to a specific position if BUILD incorporated the marker at one specific position only (threshold LOD = 3). If multiple positions were found, the best position was based on three criteria: (1) if the sequence of the contig in which the marker was located showed a (partial) BLAST hit against one of the possible locations indicated by BUILD, (2) if one of the positions in the BUILD output had a higher LOD score (>1) than all other positions and, (3) if a gap was found between two (super) contigs on the physical map. If no specific position was found using these criteria, the marker was excluded from the analysis. The BUILD output was, furthermore, used to determine potential errors in the marker order. Markers that showed high recombination rates compared to flanking markers (>3 cM on both sides) were taken out of the map and reanalyzed by BUILD. CHROMPIC was used to identify double recombinants, which, at the marker density used, are a good indication of marker order errors or genotype errors. Double-recombinant markers were reanalyzed by BUILD to determine the most likely position. Double recombinants that could not be resolved after repositioning were most likely caused by genotyping errors, and were therefore removed from the dataset. FLIPSN (*n *= 5) was also used to correct errors in the marker order. If an alternative marker order was more likely than the initial one (LOD increased by >1), the new marker order was used. To decrease errors and increase the accuracy of the map, the CHROMPIC, BUILD, and FLIPSN options were used repeatedly for each chromosome until no double recombinants were observed and the most likely marker order was achieved for the remaining markers. Finally, the FIXED option was used to construct the sex-specific and sex-average linkage maps. For the markers that remained in the chromosome unassigned, TWOPOINT analyses were performed to find linkage between the markers (LOD = 3).

### Recombination rate

Recombination rates were calculated for nonoverlapping bins of approximately 500 kb. Linkage maps for population 1 and 2 were constructed with all of the markers that were informative in at least one of the populations. The recombination rate of each bin is expressed as the genetic length in centimorgans divided by the genomic length in mega base pairs.

### Statistical Analysis

To test if differences in map distances between populations differed significantly we assumed that 1 cM equals a recombination fraction of 0.01 and calculated the Z-test statistic as

where

θ_1 _= the recombination fraction in population 1,

θ_2 _= the recombination fraction in population 2,

n_1 _= the average number of informative meiosis in population 1,

n_2 _= the average number of informative meiosis in population 2.

p-values were obtained from a standard normal distribution. Recombination fractions were determined for sliding windows consisting of eight bins. When differences in recombination fractions between males and females were tested, it was assumed that both sexes contributed equally to the number of informative meiosis. We considered a nominal p < 0.01 as suggestive evidence for differences in recombination fraction. Further, for results to be significant, a more stringent significance criteria p < 2.46*10^-4 ^was defined that accounts for multiple testing along the genome. Multiple testing was accounted for by applying a Bonferroni correction assuming 203 independent tests and a nominal α = 0.05. For in total 1624 "windows" differences in recombination were determined, however, as a result of the sliding window approach (a window consisting of eight bins), every 8^th ^sliding window is truly independent which results in 203 independent tests.

## Results

### Linkage analysis

In total, 13,340 informative markers (75% of all markers on the SNP beadchip) and 1,619 individuals were used to construct the combined linkage map of the two populations (Additional Files 2 and 3). In total, 613 markers that had not been mapped to a known chromosome or linkage group were included on the beadchip. Of the 613 unassigned markers, 103 did not pass quality control, 150 were homozygous, and 360 were informative (Additional Files 3 and 4). Of the informative markers, 343 could be assigned to a known chromosome or linkage group, and 17 could not. These 17 markers also showed no linkage to each other, even when the LOD score threshold was set to 2. From the 343 markers that were assigned to a known chromosome with AUTOGROUP, 230 were included in the final linkage map. No specific position on a chromosome could be determined for the remaining 110 markers (three GGW assigned markers were not included in the analysis), and they were therefore not included in the linkage map.

As a starting point for building the linkage map, we used the marker order based on the position of the markers on the sequence map. In general, this order appeared to be in agreement with the most likely marker order for the linkage map. Some adjustments, nevertheless, were made: on GGA5, a block of thirteen markers was inverted, which resulted in a 1.4 cM decrease in the size of the map; on GGA13, five markers were inverted, which resulted in a 1.5 cM decrease in the size of the map; in linkage group LGE22, rearranged markers resulted in a decrease of 3.1 cM; and in linkage group LGE64, rearranged markers resulted in a decrease of 9.4 cM. The number of informative meioses per mapped marker for the combined linkage map ranged from 20 to 1,242, with an average of 517. The total length of the sex-average map was 3,053.5 cM (Table 1). The female sex-specific map was 211.5 cM smaller than the male sex-specific map, with a female-to-male ratio of 0.93. On average, the recombination rate of the combined map was 3.0 cM/Mb. The average recombination rate decreased as the length of the chromosome increased; for the macrochromosomes, a lower recombination rate (about 2 cM/Mb) was observed compared to the microchromosomes (3-14 cM/Mb)

**Table 1:** The linkage map lengths and recombination rates for the chicken chromosomes of the combined populations.

	**Length**^1^	Sex-average	Sex-specific		Recombination rate
Chromosome			Female	Male	
	(Mb)	(cM)	(cM)		(cM/Mb)
GGA1	200.9	413.5	377.1	455.3	2.1
GGA2	154.8	281.3	259.9	303.5	1.8
GGA3	113.6	236.9	225.6	250.2	2.1
GGA4	94.2	195.2	182.5	207.7	2.1
GGA5	62.2	154.4	154.9	155.1	2.5
					
GGA6	37.3	93.8	85.0	102.4	2.5
GGA7	38.3	103.1	99.0	107.3	2.7
GGA8	30.6	96.6	94.2	98.9	3.2
GGA9	25.5	88.1	85.4	91.1	3.5
GGA10	22.5	80.6	79.6	81.1	3.6
					
GGA11	21.9	64.0	63.3	64.9	2.9
GGA12	20.5	69.1	67.9	70.7	3.4
GGA13	18.9	62.7	63.8	61.6	3.3
GGA14	15.8	67.4	72.5	65.2	4.3
GGA15	13.0	53.6	52.9	54.2	4.1
					
GGA16	0.4	55.6	59.1	53.5	n.d.^2^
GGA17	11.2	50.9	51.5	51.0	4.6
GGA18	10.9	51.7	49.9	53.5	4.7
GGA19	9.9	52.3	53.2	52.0	5.3
GGA20	13.9	55.1	55.2	54.8	4.0
					
GGA21	6.9	56.9	57.2	56.5	8.2
GGA22	3.9	56.4	59.9	52.4	14.3
GGA23	6.0	52.3	51.4	53.0	8.7
GGA24	6.4	53.2	53.4	52.4	8.3
GGA25	2.0	57.1	54.0	59.4	n.d.^2^
					
GGA26	5.1	52.3	51.4	52.9	10.3
GGA27	4.7	51.0	50.6	51.5	10.8
GGA28	4.5	53.6	52.5	54.3	11.9
LGE22	0.9	59.3	58.5	64.5	n.d.^2^
LGE64	0.017	8.4	6.7	8.7	n.d.^2^
					
GGZ	74.6	227.7	-	227.1	3.0

Total autosomal	956.9	2,826.4	2,728.1	2,939.6	3.0
Total length	1,031.5	3,053.5	2,728.0	3,166.7	3.0

1) Physical length of the chromosome was based on the position of the last marker in the WASHUC2 build. 2) n.d. = not determined, as the chromosome showed clear evidence of sequence gaps.

To study the populations separately, linkage maps were calculated for both populations independently (Table 2). The linkage map for population 1 consisted of 12,617 markers (95% of the markers used in the combined map) (Additional File 2), and included 306 animals in 42 full- and half-sib families (*n *= 7-13 per family). The number of informative meioses per mapped marker for population 1 ranged from 20 to 231, with an average of 120. The total length of the sex-average map of population 1 was 3,498.6 cM (Table 2). The female sex-specific map was 211.8 cM smaller than the male sex-specific map, with a female-to-male ratio of 0.93. The linkage map of population 2 consisted of 9,803 markers (73% of the markers used in the combined map) (Additional File 2), and included 1,313 animals in 68 full- and half-sib families (*n *= 6-43 per family). The number of informative meioses per mapped marker for population 2 ranged from 20 to 1,118, with an average of 551. The total length of the sex-average map of population 2 was 2,812.3 cM (Table 2). The female sex-specific map was 198.6 cM smaller than the male sex-specific map, with a female-to-male ratio of 0.93, which was similar to population 1.

**Table 2:** The linkage map lengths and recombination rates for the chicken chromosomes of the two separate populations.

		Population 1				Population 2			
			
Chromosome	Length^1^	Sex-average	Sex-specific				Sex-specific		
			Female	Male	Recombination rate	Sex-average	Female	Male	Recombination rate
	(Mb)	(cM)	(cM)		(cM/Mb)	(cM)	(cM)		(cM/Mb)
GGA1	200.9	504.0	471.0	541.6	2.5	387.1	351.8	428.0	1.9
GGA2	154.8	341.4	321.1	363.5	2.2	267.7	245.9	289.4	1.7
GGA3	113.6	288.8	269.5	309.2	2.5	224.8	215.6	236.4	2.0
GGA4	94.2	237.6	227.5	247.3	2.5	183.7	171.5	196.5	1.9
GGA5	62.2	176.8	175.7	178.5	2.8	148.6	149.1	149.6	2.4
									
GGA6	37.3	110.5	97.9	122.2	3.0	89.8	81.6	97.3	2.4
GGA7	38.3	117.1	119.7	118.3	3.1	99.5	93.7	105.2	2.6
GGA8	30.6	107.5	103.1	111.3	3.5	94.0	91.9	95.6	3.1
GGA9	25.5	97.1	99.0	95.9	3.8	85.2	81.5	88.8	3.3
GGA10	22.5	94.5	91.6	97.9	4.2	75.4	73.7	76.3	3.4
									
GGA11	21.9	87.1	86.8	87.7	4.0	58.8	58.4	59.6	2.7
GGA12	20.5	89.0	90.3	88.5	4.3	64.3	62.6	66.6	3.1
GGA13	18.9	74.1	76.7	71.6	3.9	58.1	58.0	58.2	3.1
GGA14	15.8	75.2	74.9	75.4	4.8	64.2	66.5	61.6	4.1
GGA15	13.0	59.7	57.0	62.0	4.6	52.3	51.9	52.4	4.0
									
GGA16	0.4	55.4	59.1	53.1	n.d.^2^	0.3	0.5	0.0	n.d.^2^
GGA17	11.2	54.6	52.4	57.3	4.9	50.2	51.6	49.3	4.5
GGA18	10.9	58.1	56.5	60.1	5.3	49.2	47.8	50.6	4.5
GGA19	9.9	49.7	52.2	47.9	5.0	52.7	53.4	52.5	5.3
GGA20	13.9	58.4	55.8	60.5	4.2	53.9	54.9	52.9	3.9
									
GGA21	6.9	58.9	56.0	61.8	8.5	56.2	57.2	54.9	8.1
GGA22	3.9	51.6	55.4	46.5	13.1	53.6	56.0	51.9	13.6
GGA23	6.0	48.4	49.1	47.8	8.0	53.1	52.2	53.9	8.8
GGA24	6.4	51.2	49.0	53.7	8.0	53.7	54.5	52.1	8.4
GGA25	2.0	57.5	56.7	58.5	n.d.^2^	57.3	54.1	59.4	n.d.^2^
									
GGA26	5.1	50.6	50.1	50.5	9.9	52.6	51.7	53.5	10.3
GGA27	4.7	49.0	47.0	51.3	10.4	51.5	52.1	51.3	10.9
GGA28	4.5	52.9	56.8	50.9	11.7	53.7	52.0	55.2	11.9
LGE22	0.9	55.6	48.5	62.0	n.d.^2^	46.9	54.4	46.0	n.d.^2^
LGE64	0.017	23.5	27.4	22.8	n.d.^2^	4.1	4.1	3.8	n.d.^2^
									
GGZ	74.6	262.8	-	262.8	3.5	169.8	-	169.8	2.3

Total autosomal	956.9	3,235.8	3,133.8	3,355.6	3.4	2,642.5	2,550.2	2,748.8	2.8
Total length	1,031.5	3,498.6	3,133.8	3,618.4	3.4	2,812.3	2,550.2	2,918.6	2.7

1) Physical length of the chromosome was based on the position of the last marker in the WASHUC2 build. 2) n.d. = not determined, as the chromosome showed clear evidence of sequence gaps.

### Recombination rate

**Figure 1:**
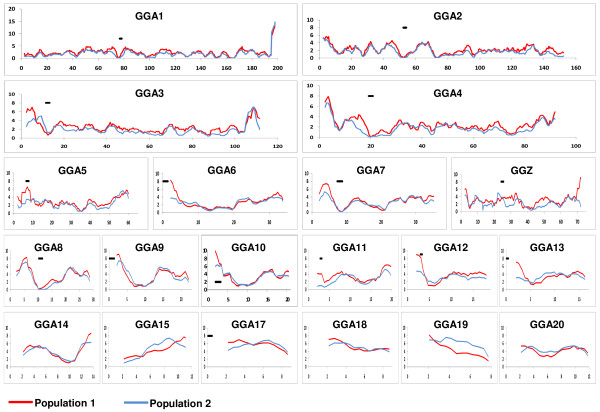
**Sex-average recombination rate for populations 1 and 2**. Recombination rate was calculated for 500 kb nonoverlapping bins, and plotted using a sliding window of eight bins. Population 1 is shown in red and population 2 is shown in blue. On the x-axis, the genomic position is given in million base pairs. On the y-axis, the recombination rate is given in cM/Mb. If known, the position of the centromer is indicated by a solid black line. GGA16, GGA21--GGA28, LGE22, and LGE64 were not included in this figure, because the graphs of these 11 small chromosomes were uninformative. Note that the scale of the y-axis of GGA1 is twice as high as for the other chromosomes.

To analyze the recombination frequency along the different chromosomes, the genome was divided into 1,819 nonoverlapping bins with an average size of 560 kb (Additional File 5). For both populations, the sex-average linkage map data were used to calculate the recombination rates of these bins (Figure 1). Recombination rates varied from 0 to 60 cM/Mb in population 1 and from 0 to 74 cM/Mb in population 2. Overall, the recombination rates observed between the two populations showed similar trends. Nevertheless, several regions were observed where the two populations diverged with regard to recombination rates (Figure 1 and Additional File 5). On GGA 6, 11, 12, and 13, these regions exceeded the stringent Bonferroni threshold when accounting for multiple testing. On these four chromosomes, the regional difference in recombination rate between the two populations was observed at the telomere of the p arm. Similar observations were made in other chromosomes where the two populations diverged with suggestive significance (p < 0.01).

**Figure 2:**
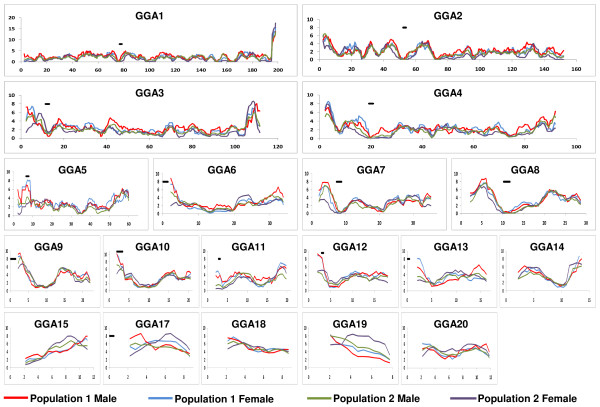
**Sex-specific recombination rate for populations 1 and 2**. Recombination rate was calculated for 500 kb nonoverlapping bins, and plotted using a sliding window of eight bins. The female map of population 1 is shown in blue, and the male map of population 1 is shown in red. The female map of population 2 is shown in purple, and the male map of population 2 is shown in green. On the x-axis, the genomic position is given in million basepairs. On the y-axis, the recombination rate is given in cM/Mb. If known, the position of the centromer is indicated by a solid black line. GGA16, GGA21--GGA28, LGE22, and LGE64 were not included in this figure, because the graphs of these 11 small chromosomes were uninformative. Note that the scale of the y-axis of GGA1 is twice as high as for the other chromosomes.

The sex-specific linkage maps enabled us to study the effect of sex on recombination. Recombination rates were calculated for nonoverlapping bins based on the recombination rates found in the sex-specific linkage maps of both populations (Figure 2 and Additional File 5). Overall, the recombination rates observed between the two sexes of the two populations showed similar trends. However, in the regions on GGA 6, 11, 12 and 13, where the recombination rate of two populations significantly diverged, this difference appeared to be caused by a difference in female recombination rate and not due to male recombination rate (Figure 2 and Additional File 5). For the regions where the two populations diverged with suggestive significance (p < 0.01), the difference in female recombination rate often exceeded the Bonferroni threshold, while there was no statistical evidence for difference in male recombination rate.

## Discussion

The high accuracy of the SNP genotyping, the large number of markers (*n *= 13,340), and the large number of animals (*n *= 1,619) resulted in a high-resolution linkage map of the chicken genome, which significantly exceeds the resolution of previously published linkage maps [7,10-12]. The current map consists of 13,340 markers, which is an increase of 43.9% compared to the latest consensus map, which comprises 9,268 markers [7]. In total, 2,819 SNP markers overlapped between the two studies. The increased marker density enabled us to efficiently detect genotype errors, thereby increasing the accuracy of the linkage map compared to the latest consensus map.

The use of a large number of animals in the current study resulted in a 6-fold increase (517 vs. 85) in the average number of informative meioses per mapped marker, thereby increasing the resolution of the current map compared to the latest published linkage map [7]. The higher resolution enabled us, moreover, to order closely linked markers. The linkage map comprises 31 linkage groups, with a total length of 3053.5 cM for the sex-average map of the combined population (Table 1). This length is comparable to previous estimates [7].

The construction of separate linkage maps for both populations enabled us to study differences in recombination between the two populations. The sex-average linkage map of population 1 (broiler × broiler cross, 3,498.6 cM) is 24.4% larger than the map of population 2 (purebred broiler line, 2,812.3 cM) (Table 2). The difference between the two populations has a biological origin, although differences in informative markers occasionally contributed to the difference between the two maps. An extreme example is GGA16; in population 1, the single marker located at the end of the chromosome (55.4 cM) was uninformative in population 2, and resulted in a chromosome length of only 0.3 cM in this population. Roughly one third of the difference between the two populations on the autosomal chromosomes is explained by the telomeric regions (defined as 10% of the chromosome length at both telomeres). A clear example is GGZ, where the difference between the two populations (93 cM) is primarily caused by the telomeric regions. In previous studies, large differences in the length of this chromosome have been reported, varying from 193 to 284 cM [7,26]. In both populations used in this study, the female specific linkage map was approximately 200 cM smaller than the male specific linkage map. However, for the sex-specific linkage map of population 1, no difference was found between female and male in the latest published linkage map. In addition to having more markers in this study, we also selected more animals and included extra generations of population 1 compared to the last published linkage map. The increased marker density, additional animals, and generations were not expected to have an influence on the (sex-specific) linkage map between the two studies. Nevertheless, the increased number of animals in the current study (and therefore the increased amount of informativity) most likely resulted in a more accurate linkage map, so that the 200 cM difference between female and male recombination could be determined. The 200 cM difference between female and male recombination is, moreover, also seen in population 2, indicating that the female map in chickens is indeed smaller.

Burt and Bell hypothesized that selection leads to high rates of recombination [16]. Although the selection criteria were based on different traits, all three lines used in this study experienced similar selection pressure (personal communication A. Vereijken of Breeding Research and Technology Centre, Hendrix Genetics). We therefore conclude that the difference in recombination between the two populations was not caused by selection pressure *per se*. The linkage map length of the purebred broiler line (population 2) was very similar to that of other chicken populations such as the East Lansing population (partially inbred Red Jungle Fowl × highly inbred White Leghorn cross) and the Uppsala population (Red Jungle Fowl × White Leghorn cross) [7]. Therefore, it appears that the broiler × broiler cross deviates from the other chicken populations by having a high recombination rate. Although not caused by selection, the high recombination rate in this cross could be the result of either a high recombination rate in one or both of the parental lines, or by as-yet unidentified genomic differences between the two lines of this cross.

The high-resolution linkage map enabled us to study recombination hotspots within the two populations and the two sexes (Figures 1 and 2). Excluding bins with apparent sequence gaps, the recombination rate for the nonoverlapping bins varied from 0 to 20 cM/Mb. This range is in agreement with previous findings in the chicken genome [7]. Overall, recombination rates tended to be similar between the two populations (Figures 1 and 2). However, when regional differences in recombination hotspots were observed between the two populations, the location of these hotspots were mainly located at the telomere of the p arm (Figure 1 and Additional File 5). Moreover, the differences in recombination rate at the telomere appeared to be caused by female-specific recombination hot spots (Figure 2 and Additional File 5). Because the broiler × broiler cross (population 1) appears to deviates from other chicken populations, as described above, we conclude that this population had an increased female recombination rate near the telomere of the p arm.

To improve the current genome build, 613 unassigned markers were included on the 18K Illumina iSelect Beadchip. At the time, we assumed that these markers would have a high likelihood of being located on one of the missing microchromosomes, or in sequence gaps that still exist in the current genome build. In total, 59% (*n *= 360) of all unassigned markers were informative in at least one of our two mapping populations. For the markers that had already been mapped to a chromosome, these values were considerably higher: 77% (*n *= 13,250). An explanation for the difference in informativity is that chromosome unassigned is known to be mainly comprised of sequences with lower quality, genome duplications and gene families (e.g. MHC). In particular, genome duplications and gene families are likely to result in the alignment of paralogous sequences, resulting in a higher frequency of false-positive SNPs. These false-positive SNPs contribute to the decreased informativity of the chromosome unassigned markers.

The majority of the informative unassigned SNPs on the beadchip were mapped in sequence gaps of chromosomes or linkage groups that were already covered by the WASHUC2 build. Only 17 SNPs did not appear to be located on any of these chromosomes; however, there was no linkage among these SNPs. The genome coverage for the microchromosomes is, therefore, not extended by the current linkage map. The fact that no new linkage groups were found is in agreement with previous findings that the sequences from the missing chromosomes may be difficult to clone and propagate in *E. coli*; therefore they are missing in the current draft sequence of the chicken genome [7,13].

In addition to the new markers that were added to improve the current genome build, the high-resolution linkage map presented in this study can be used to correct mistakes in the order of sequences in the current genome assembly. A marker order in the linkage map that is different from the physical map may indicate mistakes in genome assembly. Although the marker order of the linkage map was mainly in agreement with the order of these markers on the physical map, some changes were observed. For the microchromosomes and the two linkage groups, these changes were not unexpected, because several of these chromosomes were known to be poorly assembled. On GGA5 and GGA13, the changed marker order suggests an incorrect genome assembly or a possible inversion in the broiler populations compared to the reference sequence (Red Jungle Fowl). In our data, the inversed marker order on GGA5 led to a 1.6 cM decrease in the length of the population 2 map 2, although no reduction in map length was seen in population 1. Similarly, on GGA13, the inversed marker order resulted in a 1.8 cM decrease in the map of population 2, but had no influence on the map length of population 1.

## Conclusions

In this study, we present a linkage map of the chicken genome at a substantially higher resolution than previously published linkage maps. The increased resolution enabled us to study underlying recombination hotspots. There were regional difference in recombination hotspots between the two mapping populations in several chromosomes near the telomere of the p arm, and sex-specific analysis revealed that these regional differences were caused mainly by female-specific recombination hotspots in the broiler × broiler cross.

## Authors' contributions

MG, RC, PA and MGE conceived and designed the experiments. PA, MGE and TV performed the experiments. MGE, PA and TV analyzed the data. MGE, MG and RC wrote the paper. All of the authors read and approved the final manuscript.

## Supplementary Material

Additional file 1 **Detailed information of all used markers used in the construction of the linkage map**. This table includes all SNPs used in the construction of the linkage map including their position on the chromosome, newly assigned chromosome if applicable, status and sequence. Click here for file

Additional file 2 **The complete linkage map**. This file contains the linkage map of the combined and separate population. It includes the sex-average and sex-specific maps. Click here for file

Additional file 3 **Overview of all used markers**. This figure shows an overview of all markers and includes the number of markers assigned, unassigned, not mapped, mapped, not used, chromosome unassigned, homozygous or rejected by Beadstudio. Click here for file

Additional file 4 **Detailed information about the chromosome unassigned markers**. This table includes all chromosome unassigned markers, whether they are assigned or mapped to a chromosome, or why they were rejected for the construction of the linkage map. Click here for file

Additional file 5 **The 500 kb bins used to study the recombination rates**. This file includes all 1819 bins that were used to study recombination rates. It includes the bins for both populations (sex-average, female- and male-specific), and the Z and p-values of the eight bin sliding windows. Click here for file
