# Supplementary figures and images for: Regional differences in recombination hotspots between two chicken populations
Source: BMC Genet. 2010 Feb 8;11:11. doi: 10.1186/1471-2156-11-11 (PMC2834597; doi:10.1186/1471-2156-11-11)

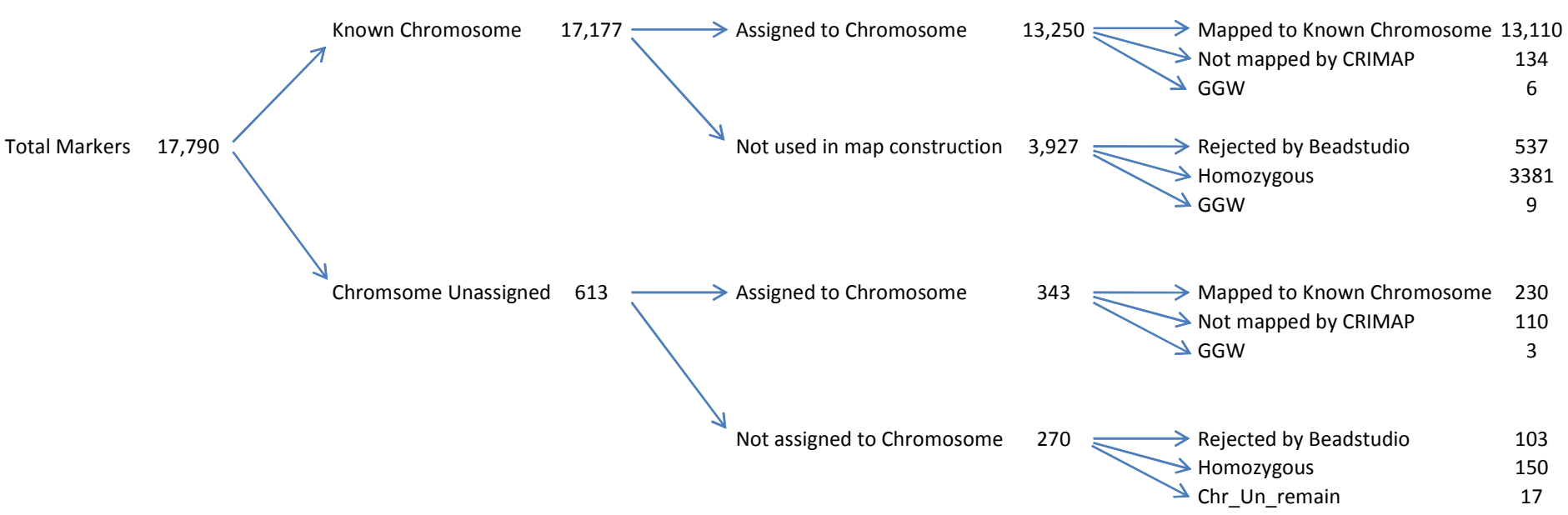

Supplement: Additional file 3 — Overview of all used markers. This figure shows an overview of all markers and includes the number of markers assigned, unassigned, not mapped, mapped, not used, chromosome unassigned, homozygous or rejected by Beadstudio. [file 1471-2156-11-11-S3.PDF]
